# Bioactive Compounds, Pharmacological Actions, and Pharmacokinetics of Wormwood (*Artemisia absinthium*)

**DOI:** 10.3390/antibiotics9060353

**Published:** 2020-06-23

**Authors:** Gaber El-Saber Batiha, Ahmed Olatunde, Amany El-Mleeh, Helal F. Hetta, Salim Al-Rejaie, Saad Alghamdi, Muhammad Zahoor, Amany Magdy Beshbishy, Toshihiro Murata, Adrian Zaragoza-Bastida, Nallely Rivero-Perez

**Affiliations:** 1 Department of Pharmacology and Therapeutics, Faculty of Veterinary Medicine, Damanhour University, Damanhour 22511, Egypt; 2 Department of Biochemistry, Abubakar Tafawa Balewa University, Bauchi 740272, Nigeria; olatundebch@gmail.com; 3 Department of Pharmacology, Faculty of Veterinary Medicine, Menoufia University, Menouf 32511, Egypt; Amany.ahmed1074@gmail.com; 4 Department of Medical Microbiology and Immunology, Faculty of Medicine, Assiut University, Assiut 71515, Egypt; helal.hetta@uc.edu; 5 Department of Internal Medicine, University of Cincinnati College of Medicine, Cincinnati, OH 45229, USA; 6 Department of Pharmacology & Toxicology, College of Pharmacy, King Saud University, Riyadh 11421, Saudi Arabia; rejaie@KSU.EDU.SA; 7 Laboratory Medicine Department, Faculty of Applied Medical Sciences, Umm Al-Qura University, P.O. BOX 715, Makkah 21955, Saudi Arabia; ssalghamdi@uqu.edu.sa; 8 Department of Biochemistry, University of Malakand, Chakdara Dir Lower, Khyber Pakhtunkhwa 18800, Pakistan; mohammadzahoorus@yahoo.com; 9 National Research Center for Protozoan Diseases, Obihiro University of Agriculture and Veterinary Medicine, Inada-cho, Obihiro, Hokkaido 080-8555, Japan; 10 Department of Pharmacognosy, Tohoku Medical and Pharmaceutical University, 4-4-1, Komatsushima, Aoba, Sendai, Miyagi 981-8558, Japan; murata-t@tohoku-mpu.ac.jp; 11 Área Académica de Medicina Veterinaria y Zootecnia, Instituto de Ciencias Agropecuarias, Universidad Autónoma del Estado de Hidalgo, Rancho Universitario Av. Universidad km 1, EX-Hda de Aquetzalpa, 43600 Tulancingo, Mexico; adrian_zaragoza@uaeh.edu.mx (A.Z.-B.); nallely_rivero@uaeh.edu.mx (N.R.-P.)

**Keywords:** traditional uses, medicinal herb, biological activities, *Artemisia absinthium*, phytochemical compounds

## Abstract

Plants have been used since ancient times to cure certain infectious diseases, and some of them are now standard treatments for several diseases. Due to the side effects and resistance of pathogenic microorganisms to antibiotics and most drugs on the market, a great deal of attention has been paid to extracts and biologically active compounds isolated from plant species used in herbal medicine. *Artemisia absinthium* is an important perennial shrubby plant that has been widely used for the treatment of several ailments. Traditionally, *A. absinthium* has always been of pharmaceutical and botanical importance and used to manage several disorders including hepatocyte enlargement, hepatitis, gastritis, jaundice, wound healing, splenomegaly, dyspepsia, indigestion, flatulence, gastric pain, anemia, and anorexia. It has also been documented to possess antioxidant, antifungal, antimicrobial, anthelmintic, anti-ulcer, anticarcinogenic, hepatoprotective, neuroprotective, antidepressant, analgesic, immunomodulatory, and cytotoxic activity. Long-term use of *A. absinthium* essential oil may cause toxic and mental disorders in humans with clinical manifestations including convulsions, sleeplessness, and hallucinations. Combination chemotherapies of *artemisia* extract or its isolated active constituents with the currently available antibabesial or anti-malarial drugs are now documented to relieve malaria and piroplasmosis infections. The current review examines the phytoconstituents, toxic and biological activities of *A. absinthium*.

## 1. Introduction

*Artemisia absinthium* L., commonly known as wormwood, is an important perennial shrubby medicinal plant native to Asia, Middle East, Europe, and North Africa [1]. *Artemisia* is one of the most predominant and widely distributed genus in Asteraceae family that is composed of more than 500 different species classified as annual, perennial, and biennial natural plants or small shrubs (Table 1) [2]. 

**Figure 1:**
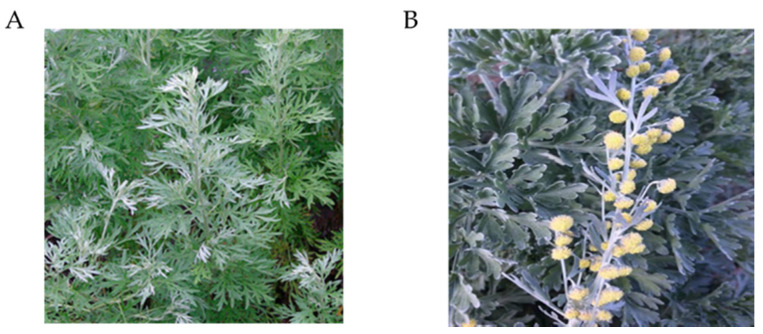
Aerial parts (**A**) and flower (**B**) of *Artemisia absinthium* [5,6].

*A. absinthium* is named by several vernacular names. It is named as green ginger, absinthe, *absinthium*, wormwood in English; Genepi in Latin; Vermouth in French; Apsinthion in Greek; *Absinthium* in Hemopathy, Anjenjo in Mexican; Yang ai, Kuai in Chinese; Majtari, Majri, Mastiyarah, Karmala in Hindi; Absinth, Wermut in German; nigayomogi in Japanese; Damseeh, and Afsanteen in Arabic [3]. *A. absinthium* root is perennial with a firm, prolonged, woody, and leafy stem and has a warm and aromatic taste. The stem is about 2–2.5 feet tall, white in color and almost covered with fine silky hairs. The leaves are white on both sides, 3 inches long and 1.5 wide with slender and unshaped segments and the leaf-stalks are slightly winged at the margin and the leaves are reduced to three, or even one linear subdivision on the flower-stalks. Flowering takes place from early summer to early autumn [4]. The flower heads are short, nearly orbicular and hang in an erect, leafy panicle, and the little flowers are pendulous with a greenish-yellow color. The leaves and flowers are very bitter, with a distinctive aroma, resembling that of thujone. Figure 1 shows the aerial parts and flower of *A. absinthium*. 

*A. absinthium* is one of the most important herbs that has exhibited several pharmacological activities, such as being antimicrobial, insecticidal, antiviral, hypoglycemic, hepatoprotective, wound healing, anti-inflammatory, and cardiovascular diseases [5]. Moreover, it has shown a broad spectrum antioxidant and anticancer activities [7,8]. The current review aims to further understand the traditional uses, beneficial and pharmacological effects of *A. absinthium* and its related compounds, as well as their pharmacokinetics and concerns around safety. 

## 2. Method

In this review article, a comprehensive search was performed in the following databases: PubMed, Web of Science, and Google scholar for studies published from 1985 to 2020. The following medical subject headings and keywords such as: “*A. absinthium*,” ‘Wormwood’, ‘Bioactive compounds’, and “Pharmacological activities” were used. We removed duplicated papers, then screened the data, ruled out irrelevant work, and then screened the full-text documents. Inclusion criteria includes a number of factors, involving original articles or review article, work on natural or chemical compounds. Although certain exclusion requirements included non-English documents, inadequate methods, and lack of access to the full text.

## 3. Bioactive Constituents 

*A. absinthium* contains many phytochemical compounds namely, lactones, terpenoids (e.g., *trans*-thujone, γ-terpinene, 1,4-terpeniol, myrcene, bornyl acetate, cadinene camphene, *trans*-sabinyl acetate, guaiazulene, chamazulene, camphor, and linalool), essential oils, organic acids, resins, tannins, and phenols [9]. It also contains flavonoids (e.g., quercitin), flavonoid glycosides such as isorhamnetin-3-*O*-rhamnose glucoside, isoquercitrin, quercitin-3-*O*-D-glucoside, quercetin-3-*O*-rhamnoglucoside, and isorhamnetin-3-*O*-glucoside, and phenolic acids (coumaric, syringic, salicylic, chlorogenic, and vanillic acids) which contribute to free radical scavenging mechanism [10]. In addition, Ahamad et al. [11] reported that methanolic *A. absinthium* extract contains isoflavone glycosides that are characterized as *Artemisia* isoflavonyl glucosyl diester and bis-isoflavonyl dirhamnoside. Previous studies documented that *A. absinthium* essential oils are rich in myrcene, *trans*-thujone, *cis*-epoxyocimene, *cis*-chrysanthenyl acetate, and *trans*-sabinyl acetate are the most common compounds found in [12,13]. The medicinal efficacy of wormwood is often based on its bioactive ingredient in the dimeric guaianolides absinthins, as it is used more effectively than other *Artemisia* species as it contains approximately 0.2% of absinthin [14,15]. In addition, fresh wormwood is considered the best source of azulene, yielding between 40 and 70 mg % of azulene [16]. Table 2 shows the main active constituents isolated from *A. absinthium*. 

## 4. Pharmacological Actions

### 4.1. Traditional Uses of A. absinthium

*A. absinthium* from different geographical locations has been of pharmaceutical and botanical importance and has been used traditionally for the management of several disorders including hepatocyte enlargement, hepatitis, gastritis, jaundice, wound healing, splenomegaly [17], dyspepsia and indigestion, flatulence, gastric pain, anemia, anorexia [18], esophageal bowel syndrome with irritation, weak memory tremors [19], depression, epilepsy, chronic fever [20], skin diseases, gout, and rheumatism [21]. Additionally, it is used as anthelmintic and insect repellents [22], as an additive source for ruminants, particularly in promoting the rumen fermentation pattern for efficient utilization of diets (Table 3). Kim et al. [23] showed that administration of dried *A. absinthium* rather than rice straw did not alter the pH of the rumen. Moreover, it has been documented to alleviate pains during labor and for the management of sclerosis and leukemia [24]. It is widely used in the food industry for the production of aperitifs, spirits, and bitters [25]. Additionally, *A. absinthium* ointment has been used externally to reduce the stiffness of muscles and joints as well as help in healing bruises [26]. Furthermore, wormwood is employed to relieve childbirth pain and it is also employed to relieve pains during the menstrual cycle [27], and for the cardiac disorder and hypertension [1,28,29].

### 4.2. Antioxidant Activity

The free radical scavenging and antioxidant activity of *A. absinthium* has been reported by Ali et al. [7]. They documented that this activity is attributed to the presence of several phenolic compounds (gallic acid, coumaric acid, vanillic acid, syringic acid, chlorogenic salicylic acid) and flavonoids including quercetin and rutin. Recently, Bora et al. [38] revealed that *A. absinthium* possesses potent antioxidant properties and its methanolic extract has clearly demonstrated neuroprotection evidenced by the reduction of lipid peroxidation level associated with decreasing thiobarbituric acid reactive substances (TBARS) level and the recovery of endogenous antioxidant (e.g., superoxide dismutase (SOD) glutathione (GSH)), indicating that *A. absinthium* may be used as a preventive agent against diseases related to oxidative stress. Another study evidenced the free radical scavenging action and cytoprotective effect of *A. absinthium* ethanolic extract against oxidative injury in fibroblast-like cells [39]. The plant extracts were tested for free radical scavenging action by estimating their capacity to inhibits 1,1-diphenyl-2-picryl-hydrazyl (DPPH) free radical and reactive hydroxyl radical during the Fenton reaction trapped by 5,5-dimethyl-1-pyrroline-*N*-oxide through the use of electron spin resonance spectroscopy [24]. Thus, *A. absinthium* was recognized to be a vital source of natural antioxidant substances.

### 4.3. Antioxidant Related Effects

#### 4.3.1. Antitumor Activity

Shafi et al. [8] have studied the antiproliferative effect of methanolic *A. absinthium* extract on estrogen-unresponsive MDA-MB-231 human breast and an estrogen-responsive MCF-7 cancer cell lines. They showed that *A. absinthium* stimulated 50% abrogation on the proliferation of MDA-MB-231 and MCF-7 cells. They reported that the anticancer mechanisms of *A. absinthium* extract was attributed to the activation of the mitogen-activated protein kinase kinase (MEK)/extracellular signal-regulated kinase (ERK) signaling pathway, which simultaneously activates the mitochondrial pathway of caspase activation, and regulates Bad and Bcl-2 family proteins, resulting in MCF-7 and MDA-MB231 cells apoptotic death [8]. Consistently, Ferreira et al. [40] showed that the aerial parts crude extract of *A. absinthium* abrogates cell proliferation and stimulates programmed cell deaths in carcinoma estrogenic-unresponsive cell line (MDA-MB-231), an estrogenic-responsive cell line (MCF-7) cell line through the mediation of intracellular signaling mode of action [41]. Some studies have shown that chlorogenic acid (5-*O*-caffeoylquinic acid) has demonstrated an inhibitory effect on carcinogenesis in the liver, large intestine, and tongue and shown protective effect on in vivo oxidative stress model [42]. Artesunate, an artemisinin derivative, demonstrated in vitro and in vivo antitumor effect [43]. Chlorogenic acid (5-*O*-caffeoylquinic acid) isolated from *A. absinthium* extract displayed an inhibitory effect on carcinogenesis in the liver, large intestine, and tongue, and demonstrated ameliorative effect on oxidative stress in vivo. Another compound (artemisetin) extracted from *A. absinthium* showed significant antitumor effect against melanoma B16, however, insignificant action was recorded on the growth retardation of Pliss lymph sarcoma [44]. Crespo-Ortiz and Wei. [45] assumed that artemisinin bioactivation takes place in the endosome after the release of iron induced by the pH from the internalized transferrin. Iron activated-artemisinin provides carbon-centered radicals that can facilitate the disruption of lysosome and ROS generation leading to mitochondrial injury, caspases activation, and cell death. Other study have also been correlated artemisinin toxicity with cytokinesis reduction, improved the oxidative stress levels, tumor invasion inhibition, migration, and metastasis [46]. Moreover, Parekh et al. [47] documented the anticancer activity of *A. absinthium* as well as artesunate and dihydro artesunate against a wide range of cancer cell lines namely, HO-8910 (ovarian cancer), KML-562 (chronic myeloid leukaemia), and HeLa (cervical cancer).

#### 4.3.2. Neuroprotective and Antidepressant Effects

Bora et al. [48] revealed that *A. absinthium* demonstrated neuroprotective effects on cerebral damage stimulated by reperfusion and may aid in the formation of endogenous antioxidant including glutathione peroxidase and SOD. Additionally, the plant promotes cognitive ability through its nicotinic and muscarinic action [19]. Consistently, *A. absinthium* ethanol extract has been shown to display anticholinesterase activity and to demonstrate protective mechanisms by preventing lead-induced neurotoxicity; and can alter the rats’ behaviour by restoring AChE and monoamine oxidase (MAO) enzymes to near normal activity [49]. The plant enhanced neuronal and glial cell alterations triggered by extreme lead poisoning in adult laboratory rats suggested ameliorative activity of *A. absinthium* in the degeneration of mercuric chloride-induced neurons [50,51]. Besides, the natural sesquiterpene dimer, caruifolin, obtained from *A. absinthium* L. substantially inhibited the arrangement of intracellular free reactive oxygen molecules, therefore, this mode of action could be linked to its neuroprotective mechanistic action [52]. Mahmoudi et al. [53] investigated the anti-depressant activity of *A. absinthium* using forced swimming and tail suspension tests. They revealed that *A. absinthium* extract had the same anti-depressant efficacy as imipramine in reducing the immobility period. This antidepressant action may be attributed to mechanistic action including inhibition of MAO, abrogation of depression, and selective serotonin reuptake inhibition as well as the possible combination of phytochemical compounds present in extract [54].

#### 4.3.3. Immuno-modulatory and Wound Healing Activities

*A. absinthium* was also reported to show activity against syndromes mediated by immunity in medicine. Notably, *A. absinthium* extracts was reported to reduce tumor necrosis factor-alpha (TNF-α) and provide synergistic action on healing in patients with Crohn’s disease [55]. Moreover, Shahnazi et al. [56] documented the immuno-modulatory effect of ethanolic *A. absinthium* extract on the function and maturation of dendritic cells (DCs). They revealed that this extract adjusts the immune stimuli toward a Th1 pattern at concentrations less than 100 µg/mL through activating CD40 expression on DCs, production of cytokine, as well as inhibiting the dendritic T cell-stimulating effect. It was shown that polysaccharides obtained from *A. absinthium* possess immunomodulatory effect through the Th1 response initiation and activation of NO synthesis [57]. Hoseinian et al. [58] indicated the wound healing activity of *A. absinthium* by triggering the curative development of the wound of Achilles tendon in rabbit. This may be related to the free radical scavenging of *β*-thujone and *β*-pinene isolated from *A. absinthium* [12].

#### 4.3.4. Hepatoprotective Effect

Mohammadian et al. [59] stated that *A. absinthium* hydroalcoholic extract administration promotes hepatic function and inhibits the concentration of oxidative stress indices. Consistently, the extract of *A. absinthium* promotes and maintains the structural morphology of the hepatocellular membrane resulting in reduced activities of aspartate aminotransferase and alanine aminotransferase in serum. Amat et al. [35] also supported the hepatoprotective effect of *A. absinthium*. The plant inhibited sleep time induced by hexobarbitone and displayed choleretic activity (bile flow and bile solids) and excretory action as well as stimulates the secretion of bile acids. Similarly, *A. absinthium* showed antioxidant action through free radical scavenging effect on hydrogen peroxide (H_2_O_2_) and DPPH. Hence, the plant could serve for decreasing liver injury and as a substitute for synthetic drugs used in the management of liver disease. Though hepatoprotective effects of this herb is probable at doses lower than 200 mg/kg [35] but at high doses, *A. absinthium* plays antioxidant and anti-inflammatory role thus induce hepatotoxicity by inflammation and oxidation due to its thujone bioactive constituent [60,61]. *A. absinthium* extracts are examined for hepatoprotective effect with acute liver toxicity which is demonstrated by decreasing lipid peroxidation and toxicity to tetrachloromethanes (CCl_4_) [62]. The bioactive molecules present in *A. absinthium* such as phenolic acids, flavonoids, sesquiterpene lactones, and tannins exhibit hepatoprotective action in vivo [35]. Gilani et al. [20] also ameliorated hepatic disorders including hepatitis and other hepatobiliary diseases. The possible mode of actions based in its hepatoprotective properties includes liver microsomal drug-metabolizing enzymes suppression, free radical scavenging activity, and/or calcium channels blockage. Consistently, dicaffeoylquinic acids and caffeoyl saw in *A. absinthium* displayed hepatoprotective effect.

#### 4.3.5. Renal and Hypoglycaemic Effects

Renal disorder is one of the most prevalent and chronic diseases associated with diabetes, which changes the level of amino acid products [63,64]. A remarkable reduction in the serum protein level was recorded in diabetic rats induced by alloxan. Insulin hormone promotes the uptake of amino acid, stimulates the production of protein, and prevents the protein breakdown [65]. The blood level of creatinine and urea serve as a biomarker of renal function and are elevated in diabetic experimental rats [66]. Farzaneh et al. [67] documented the efficacy of wormwood extract to reduce the destructive effect of azathioprineon on kidney tissues especially on glomerulus, malpighian body, and urine collecting ducts. Moreover. Kharoubi et al. [68] stated that aqueous wormwood extract showed antioxidant activity and protect kidney and liver from the toxicity caused by lead by restoring the Na+-K+-ATPase, Ca++-ATPase, and Mg++-ATPase levels to normal. This action may be linked to high contents of total phenolic compounds and total flavonoids as well as antioxidant effect of wormwood extract that contribute in improving renal tissue in rats-treated azathioprine as well as lead-induced oxidative damage in liver and kidney. A recent report documented the ability of different *A. absinthium* extracts in improving the kidney dysfunction parameters by enhancing serum protein and diminishing the urea and creatinine levels in diabetic rats induced by alloxan administration [69]. Krebs et al. [70] proposed that *A. absinthium* free with thujone can be used as in the management of proteinuria in patients with immunoglobulin A (IgA) nephropathy. Daradka et al. [69] reported data about the hypoglycemic activity of different concentrations of *A. absinthium* ethanolic extracts on alloxan-induced diabetic rats. They reported that 500 mg/kg and 1000 mg/kg doses of the extracts resulted in higher hypoglycemic effect than 250 mg/kg. This action maybe because of the presence of natural active compounds which include thujyl alcohol, α- and β-thujones, azulenes, cadinene, bisabolene, sabinene, phellandrene, and pinene, the main compound of this medicinal plant, which also affects insulin-sensitizing properties. It was reported that this compound, thujone, has an effective insulin-sensitizing action as it can elevate insulin-activated glucose transporter by stimulating adenosine monophosphate-activated protein kinase (AMPK), which mainly activated the translocation of insulin-stimulated glucose transporter type 4 (GLUT4) to the cell surface. Additionally, Hassan et al. [71] validated antihyperlipidemic and hypoglycemic action of the plant in type-2 diabetic patients with hyperlipidemia.

### 4.4. Biological Activity of A. absinthium and Its Related Compounds

#### 4.4.1. Anti-inflammatory and Antisnake Venom activity

Several reports have shown that *A. absinthium* and its extracts possess a significant anti-inflammatory action and this action may be attributed to its secondary metabolites including flavonoids and sesquiterpene type compounds [26,72]. These compounds exhibit their anti-inflammatory activity through inhibition of inflammatory regulators such as bradykinins, histamine, prostaglandins, and serotonin. Moreover, Ahmad et al. [73] revealed that methanolic *A. absinthium* extract resulted in different level of anti-inflammatory activity when administered at 300, 500, and 1000 mg/kg concentrations. Moreover, methanolic *A. absinthium* extract displayed a delayed anti-inflammatory response which may be caused by the plant extracts’ delayed absorption. Nalbantsoy et al. [74] investigated the inhibitory activity of *A. absinthium* methanolic extract on carrageenan-induced acute inflammation in rats. They observed that methanol extract of *A. absinthium* ameliorated the inflammation caused by snake venom. In addition, Lee et al. [75] documented the in vitro and in vivo anti-inflammatory effect of 5,6,3′,5′-tetramethoxy 7,4′-hydroxyflavone obtained from *A. absinthium*. They reported that 5,6,3′,5′-tetramethoxy 7,4′-hydroxyflavone have anti-inflammatory action by suppressing the expression of proinflammatory mediators such as inducible NO synthase (iNOS), prostaglandin E(2) (PGE(2)), nitric oxide (NO), cyclooxygenase-2 (COX-2), nuclear factor-kappaB (NF-kB) in RAW 264.7 cells stimulated with lipopolysaccharide (LPS). It also inhibited the tumor necrosis factor-α (TNF-α) serum level in collagen-treated mice. Consistently, cardomonin obtained from *A. absinthium* extracts displayed inhibited both NO release and iNOS expression by its direct effect on transcription factor binding to deoxyribonucleic acid (DNA). [76]. Zeng et al. [52] also showed that natural sesquiterpene dimer, caruifolin D found in *A. absinthium* showed high anti-neuroinflammatory action and proposed to be serve as a lead for the development of drugs for treating neuro-inflammation-related disorders. Choi et al. [77] showed that flavone isolated from *A. absinthium* inhibited interleukin-10 (IL-10) synthesis and displayed anti-inflammatory actions on cytokine, hence reducing arthritis induced by collagen in experimental mice [78].

#### 4.4.2. Antipyretic and Analgesic Activities

*A. absinthium* was reported to display an antipyretic action, Khattak et al. [79] showed that chloroform, hexane, and aqueous *A. absinthium* extracts have antipyretic action compared to the action displayed by aspirin in subcutaneous yeast inoculations in experimental rabbits. Additionally, no adverse effect was observed after *A. absinthium* treatment up to dose of 1.6 g/kg. In another work, *A. absinthium* ethanol extract containing 24-β-ethyl *p*-cholesta-7, 22-dien-3 Bat, demonstrated antipyretic action in rats with less side effect [80]. Zeraati et al. [81] revealed that *A. absinthium* extracts demonstrate a topical antinociceptive action in experimental mice. Additionally, the topical application of an ointment containing the plant ameliorates clinical symptoms from individuals affected by osteoarthritis on the knee [21].

#### 4.4.3. Cardiovascular Activity

Hurrell et al. [82] revealed that *A. absinthium* extract displayed hypolipidemic, antiatherosclerotic, and hypocholesterolemic activity. Daradka et al. [83] also investigated the lipid-reducing action of *A. absinthium* in rabbits with hypercholesterolemia. They showed that ethanol extract of *A. absinthium* decreases triacylglycerol and serum cholesterol by 8-3.5-fold. Some of the proposed lipid-lowering action of the plant is attributed to its cholestatic action in the liver through the breakdown or removal of lipoproteins and/or abrogation of lipid hydrolytic enzymes in the lysosomes secreted by the hepatocytes. Moreover, Daradka et al. [69] demonstrated the efficacy of *A. absinthium* in reducing the total cholesterol level in diabetic rats through inhibition the activities of the enzymes involved in the cholesterol biosynthesis and decreasing the lipolysis which is under the influence of insulin. Moreover, Khori et al. [84] showed that *A. absinthium* extracts have antiarrhythmic action during supraventricular tachyarrhythmia treatment.

#### 4.4.4. Growth Performance and Hormonal Effects

Sadoughi et al. [85] recorded the efficacy of aqueous *A. absinthium* extract in diminishing the serum levels of inflammatory cytokines and elevating the efficacy of the ovary tissue antioxidant enzymes. Additionally, it has a significant enhancement of hormones like luteinizing hormone (LH), estradiol, and testosterone in rats suffering from polycystic ovary disorder. Wormwood extract activity was attributed to its bioactive constituents, phytosterols, which suppress the efficacy of 5-alpha-reductase enzyme, resulting in significant suppression on the plasma level of the dihydrotestosterone. In addition, phytosterols were documented to decrease tissue response to androgens besides diminishing the efficacy of androgens (e.g., testosterone) through suppressing aromatase and 5-alpha-reductase enzymes. Kostadinović et al. [86] recorded that the dietary supplementation of *A. absinthium* induced maximum growth performance and antioxidative status. There was a remarkable elevation in both protein and breast meat content while decreased fat content. So, *A. absinthium* can be applied as a natural feed additive for broilers and increasing the meat yield in chicken’s breast, and thus it has a significant role in promoting animal growth.

#### 4.4.5. Antiulcer and Digestive Activities

In dyspeptic conditions such as gall bladder disease and gastritis, *A. absinthium* was reported to exhibit an ameliorative effect [87,88]. Another study reported that hexane, ethanol, methanol, and chloroform extracts of *A. absinthium* displayed antiulcer properties in ulcerogenic rats induced by acetylsalicylic acid. The plant performed this action by significantly decreasing ulcer index, the gastric juice volume and reducing the activity of peptidase enzyme [87]. Consistently, Azizi et al. [89] reported that oral administration of *A. absinthium* ethanolic extract showed antiulcer activity in BALB/C mice by enhancing palatability and thus affecting feed intake. Kim et al. [90] indicated that an 18% increase in the intake of organic matter and dry matter diets containing wormwood extract by sheep was recorded compared to sheep fed on the diet without wormwood. Moreover, Kim et al. [23] reported that the higher intake of animal diets containing silage of wormwood compared to the control diet (without wormwood silage) in sheep, which characterized by a higher concentration of protein, lower fiber and higher rate of digestibility. Moreover, Kreitmair. [91] reported the efficacy of *A. absinthium* extracts or teas for the treatment of gastrointestinal tract disorders due to its content of bitter substances and essential oil. He documented that this action depends mainly on enhancing the bile production and secretion. Clinical studies reported that ethanolic *A. absinthium* extracts can increase gastric, biliary, and intestinal secretion in humans after oral administration and this effect may be due to its content of essential oil and bitter substances [80]. The pharmacological activities of *A. absinthium* and its related compounds are summarized in Table 4.

### 4.5. Activities Related to Infectious Diseases

#### 4.5.1. Antibacterial Activity

Studies have shown the wide-spectrum inhibitory effect of *A. absinthium* against several microorganisms and this was attributed to its essential oil compositions. *A. absinthium* ethanol extracts abrogate *Staphylococcus aureus* (ATCC 29213) strain with inhibition zones 10–15 mm in diameter, however, not shown antibacterial potential against *Escherichia coli* DM, *Streptococcus faecalis*, and *Bacillus subtilis* var. *niger* ATCC 10 [92]. *A. absinthium* extracts demonstrated effective antimicrobial action particularly against Gram-positive pathogenic bacteria [93]. Sengul et al. [94] demonstrated that methanolic *A. absinthium* extract resulted in inhibitory effect against *B. subtilis* ATCC 6633, *Salmonella typmiruim* RSSK 95091, *B. cereus* 6230, *S. thermophilus* 6453, *Providencia alcalifaciens* 3215, and *Pseudomonas putida* 1617 higher than those of ofloxacin and novobiocin. Another study showed that the topical application of a hydroalcoholic *A. absinthium* extract at the site of the infected wound revealed remarkable antibacterial action on *S. aureus*. The antimicrobial action recorded may be because of synergistic action between the minor (e.g., α-pinene, β-pinene) and major (e.g., camphor, *p*-cymene, caryophyllene) compounds in *A. absinthium*. The mechanism by which *A. absinthium* employed its antimicrobial activity is attributed to bioactive components (monoterpene hydrocarbons) including α-pinene, caryophyllene, camphor, β-pinene, and *p*-cymene, as these biomolecules were reported to permeabilize the biological membranes interfere with fluidity of membranes [95]. Juteau et al. [96] stated that the antibacterial action of essential oil extracted from *A. absinthium* was significantly higher than gentamicin against *S. aureus* (sensitive and resistant strains), *Sal. typhi*, *E. coli* ATCC 8739, *Proteus vulgaris*, *Klebsiella pneumoniae* 10031, and *P. aeruginosa* 9027, and thus, they can be used as a natural preservative in food and pharmaceutical industries. The antibacterial action of the essential oil composition of *A. absinthium* was linked to the presence of α-phellandrene and chamazulene which were the main components of the essential oils [12,97]. Zanousi et al. [98] identified β-Thujone, *p*-cymene 1,8-cineole, *cis*-chrysanthenol, sabinene, camphor caryophyllene, and α-phellandrene as major compounds in *A. absinthium* essential oil. Mihajilov-Krstev et al. [99] documented that the minimal inhibitory concentration (MIC) of the *A. absinthium* essential oil was ranged from <0.08 mg/mL on *S. aureus* 25 923 and *P. aeruginosa* 9027 extracted from wounds and on *Enterobacter aerogenes* and *P. mirabilis* extracted from human stools to 2.43 mg/mL on *Klebsiella oxytoca* isolated from stools, whereas the minimal bactericidal concentration (MBC) of the essential oil was ranged from 0.08 mg/ mL on *Kl. oxytoca* and *S. aureus* isolated from wounds and *E. aerogenes* isolated from stools to 38.80 mg/mL on *Listeria monocytogenes* ATCC 7644. Besides, essential oils from *A. absinthium* were reported to inhibit *Listeria monocytogenes*, *S. aureus* and *B. cereus* with MIC of 0.14, 0.62, and 0.8 μL/mL, respectively and they act by suppressing the biosynthesis of proteins, RNA, DNA, and polysaccharide in the bacterial cells [100]. Recently, Bartkiene et al. [101] investigated the antimicrobial activity of the *A. absinthium* water extract and essential oil, lactic acid bacteria strain, *Lactobacillus uvarum* LUHS245, and blackcurrants juice preparation BY against *Kl. pneumoniae*, *Salmonella enterica*, *P. aeruginosa*, *Acinetobacter baumanni*, *Proteus mirabilis*, methicillin-resistant *S. aureus* (MRSA) M87fox, *Enterococcus faecalis*, *Enterococcus faecium* 103, *B. cereus* 18 01, *Streptococcus mutans*, *Enterobacter cloacae*, *Citrobacter freundii*, *Staphylococcus epidermidis*, *Staphylococcus haemolyticus*, *Pasteurella multocida*. They revealed that *Lactobacillus uvarum* LUHS245 strain inhibited 14 from the 15 tested pathogenic strains, and the highest inhibition zones against *Pasteurella multocida* and *B. cereus* 18 01 were found (22.0 ± 0.2 and 21.5 ± 0.3 mm, respectively). *A. absinthium* water extract showed antimicrobial activity against *Pasteurella multocida* with MIC value of 20.4 ± 4.1 mm, however *A. absinthium* essential oil at 0.1% concentration inhibited *Staphylococcus epidermidis*, MRSA, *Pasteurella multocida*, *B. cereus*, *Streptococcus mutans*, and *Enterococcus faecium*. They concluded that *A. absinthium* essential oil, lactic acid bacteria strain LUHS245, and blackcurrants juice formulation immobilized in agar is the best one that consisted of all these and this formulation showed higher total phenolic compounds content, as well as higher overall acceptability.

#### 4.5.2. Antiviral Activity

Interestingly, Anwar et al. [102] reported the antiviral effect of *A. absinthium* extract against viral hepatitis and this effect was due to the presence of several bioactive compound to inhibit the integrase enzyme from human immunodeficiency virus (HIV-1) from connecting the DNA from the host cell with the reversibly transcribed viral DNA. They mentioned that *A. absinthium* relief 80–90% symptoms from viral hepatitis. Ansari et al. [103] revealed that the oral administration of *A. absinthium* extract to 30 patients with HBeAg-negative or positive chronic hepatitis B for 12 weeks showed potential antiviral effect against hepatitis B virus (HBV) by inhibiting DNA of hepatitis B, hepatitis B surface antigen, hepatitis B antigen, and normalized alanine transaminase in a significant fashion with no substantial adverse effects.

#### 4.5.3. Antiprotozoal Activity

*A. absinthium* and its extracts have antiprotozoal activities. The extracts of the plant exhibited antiprotozoal action on several apicomplexan parasites (e.g., *Eimeria*, *Plasmodium*, *Toxoplasma*, *Babesia*, and *Theileria*) and other protozoan parasites (e.g., *Trypanosoma cruzi*, *T. brucei*, and *Leishmania infantum*) [2,104,105]. Coccidiosis is one of the most important infections in livestock caused by *Eimeria* species and of greater economic importance due to high morbidity rates. Anticoccidial activity of *A. absinthium* extracts has been reported in ruminants as well as in poultry; however, the activity depends on the number of oocysts and the type of *Eimeria*. Aqueous *A. absinthium* extract at a dose of 3 mg/kg of feed per day induced a substantial reduction in the number of oocysts in broiler infected with *Eimeria tenella* and can be used as prophylactic treatment for moderate coccidiosis [106,107]. Nozari et al. [105] reported the potent antiparasitic effect of *A. absinthium* extract and showed that 100% tachyzoites were killed at 50, 100, and 200 mg/mL concentrations of the extract. The antileishmanial action of *A. absinthium* has been reported due to its flavonoids composition, and the activity of which is based on the existence of two hydroxyl groups at carbon position of C-4 and C-3 in the compound. Besides, the oxygenated monoterpenes present in *A. Absinthium* essential oils shows antileishmanial activity on axenic amastigote and against promastigote types of two *Leishmania* strains (*L. donovani* and *L. aethiopica*) [108]. Additionally, Bailen et al. [109] reported that *A. absinthium* essential oils demonstrated antiparasitic action on *L. infantum* at all the investigated concentrations, however, at concentrations of 400 and 800 μg/mL, the essential oils showed activity against *T. cruzi* under in vitro experimentation. Leishmanicidal and trypanocidal activities of *A. absinthium* essential oils have also been reported by Martínez-Díaz et al. [110]. Additionally, *A. absinthium* L. essential oil demonstrated antileishmanial activity against intracellular amastigotes and promastigotes in a BALB/c mouse model of experimental cutaneous leishmaniasis [111]. In a drug delivery system of essential oils with nanoencapsulation, the stability and volatility of the used essential oils were elevated and reduced, respectively and also, to sustain the efficacy and water solubility of the essential oil-based formulations to acceptable therapeutic efficiency [112]. Tamargo et al. [112] inspected that *A. absinthium* essential oils formulated in nanocochleates lead to tolerable, stable, and effective antileishmanial formulation with elevated action compared to treatment with the free essential oil. The nanocochleates *A. absinthium* essential oil demonstrated activity on non-infected peritoneal macrophage and *L. amazonensis* intracellular amastigotes with an IC_50_ of 27.7 ± 5.6 and 21.5 ± 2.5 µg/mL. Mice treated with nanocochleates *A. absinthium* essential oil (4 days/30 mg/kg/intralesional route/4 times) indicated no sign of weight loss or death. While the *A. absinthium* essential oil nanocochleates formulations decreased the disease in model of murine experimental animals by about 50%. Percentage recorded was higher than the results obtained experimental animals administered with free *A. absinthium* essential oil and control group. *A. absinthium* displayed antimalarial action as reported by some researchers [113,114]. In a four day study, alcohol and aqueous *A. absinthium* leaf extracts demonstrated a schizonticidal action on chloroquine-sensitive *Plasmodium berghei* in mice. Intraperitoneal, subcutaneous and oral treatment with both alcohol and aqueous *A. absinthium* extract showed parasitemia suppression, while the best result was obtained with the alcohol extract at a dose of 74 mg/kg (orally) [114]. The antimalarial actions of the compounds extracted from the plant were reported [115]. The aqueous extract and fraction of sesquiterpene lactone obtained from *A. absinthium* abrogated *P. falciparum* activity [104], as well as another study revealed that *A. absinthium* aqueous extract possessed significant inhibition of 89.9% [116]. The antimalarial action of sesquiterpene lactone, artemisinin, isolated from *Artemisia* extract involves stimulation of both heme and mitochondrial-mediated degradation cascade that led to lipid peroxidation resulting in cytotoxic effect through reactive oxygen species synthesis and depolarization of both cell membrane and mitochondria [117]. On the other side, the plant stimulates free radical scavenging in the heme-mediated cascade [118]. Additionally, artemisinin act on *P. falciparum* translationally controlled tumor protein (*Pf*TCTP), as it binds with the protein and form a covalent interaction leading to impaired the *Pf*TCTP activity [119,120]. Eckstein-Ludwig et al. [121] indicated that artemisinin can also inhibit *Pf*ATP6, an endoplasmic reticulum/orthologous Sarco ATPase- Ca^+2^ (SERCA) found in the cytosol. In another study artemisinin inhibited enzymes that are involved in vital metabolic cascade. These enzymes include L-lactate dehydrogenase (LDH), *S*-adenosyl-methionine synthetase (SAMS), pyruvate kinase (PyrK), spermidine synthase (SpdSyn) and ornithine aminotransferase (OAT) and it forms a covalent bond with these enzymes resulting in irreversible inhibition of their activities [122].

#### 4.5.4. Anti-fungal Activity

Reports have shown that *A. absinthium* displayed antifungal action and this could be linked to their essential oil. The antifungal action shown by essential oils present in *A. absinthium* makes the plant an essential natural product in pharmaceuticals, cosmetics, and food industries. Juteau et al. [96] reported that Croatian *A. absinthium* essential oil belongs to (*Z*)-epoxyocimene and β-thujone chemotype, while French *A. absinthium* essential oil belongs to (*Z*)-epoxyocimene and chrysanthenyl acetate chemotype. The Croatian chemotype exhibited higher fungicidal effect against *Candida albicans* and *Saccharomyces cerevisiae* var. *chevalieri* than the French one that is free from thujone, suggesting the significant role of thujones in the antimicrobial effect of *A. absinthium* essential oil. Moreover, Msaada et al. [123] revealed that Uruguay *A. absinthium* essential oil was rich in thujone and exhibited antifungal effects against *Botrytis cinerea* and *Alternaria* sp., while Turkish *A. absinthium* essential oil contains camphor, 1,8-cineole, and chamazulene as main components and showed fungicidal effect against 34 species of fungi such as *Fusarium oxysporum* and *F. solani*. Another study documented that *A. absinthium* essential oil exhibited potent antifungal effect on *Aspergillus flavus*, *A. niger*, *Epidermophyton floccosum*, *Trichophyton mentagrophytes*, *Microsporum canis*, *Candida neoformans*, and *C. albicans* with significant increase in inhibition zones with zone diameter range of 13–25 mm. and MIC was 50–100 µg/mL [124]. Whereas another study by Joshi et al. [125] stated that *Micrococcus luteus* was more susceptible to *A. absinthium* essential oil with an MIC value of 25  ±  4 µg/mL, followed by *M. flavus*, *Bacillus subtilis*, *Penicillium chrysogenum* and *A. fumigatus* with MIC values of 58  ±  8, 65  ±  8, 84  ±  15, and 91  ±  13 µg/mL, respectively. Consistently, essential oils obtained from *A. absinthium* displayed inhibitory action on three phytopathogenic fungi (*F. culmorum*, *F. graminearum*, and *F. oxysporum*) [123]. Recently, *A. absinthium*-silver nanoparticles (*A. absinthium*-Ag NPs) nanoparticles have been characterized for its fungicidal activity against three pathogenic yeasts of the *Candida* genus. They yielded lower MIC and MFC values than those shown by Ag NPs, indicating that the bioactive compounds found in *A. absinthium* synergistically increased the antifungal activity of Ag NPs [126].

#### 4.5.5. Anthelmintic Activity

*A. absinthium* has been reported as the effective natural product used as a substitute for synthetic agents in the management of diseases in animals and humans caused by parasites. Both α and β form of thujone in volatile oil obtained from *A. absinthium* have been shown to display actions against helminths [127]. Comparing with albendazole, a synthetic antihelminthic agent, aqueous and ethanol extracts of aerial parts of *A. absinthium* showed greater activity on gastrointestinal nematodes called *Haemonchus contortus* worms [128]. Additionally, Mravčáková et al. [129] showed that *A. absinthium* aqueous leaf extract was strongly active against *Haemonchus contortus* in sheep. The plant was shown to contain high flavonoids and sesquiterpene lactones, which have been stated to be responsible for the antihelminthic action with a low level of toxicity [130]. This outcome was in line with other studies revealed that *A. absinthium* ethanol extract remarkably decreased juvenile (L3) larval motility and development of egg of *Ascaris suum* in an in vitro model [112]. Consistently, Caner et al. [60] showed that treatment with methanol *A. absinthium* aerial parts extract decreased *Trichinella spiralis* larvae numbers in the muscle, quadriceps, diaphragm, tongue, and biceps-triceps rats muscles. Similarly, essential oils from the plant resulted in about a 66% decrease of adult *T. spiralis* parasites in mice intestine [131].

#### 4.5.6. Insecticidal Effect

**Figure 2:**
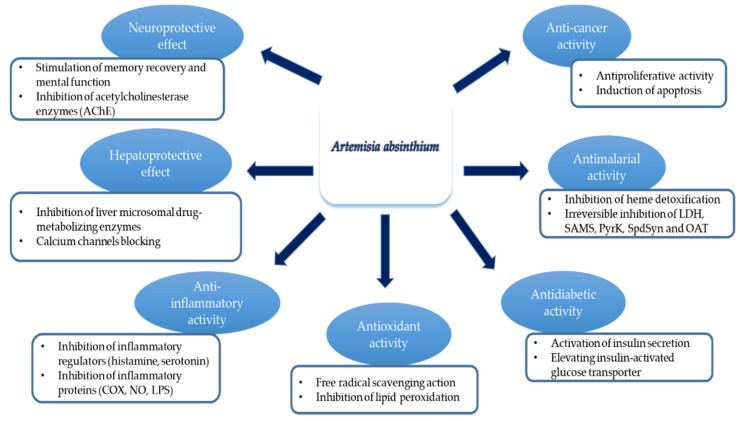
Schematic representation of different pharmacological activities of *A. absinthium* and their mechanisms.

*A. absinthium* was reported to display insect repellent action [132]. The characteristic odor of the plant makes it essential as an insect repellent and this action is linked to absinthin (sesquiterpene lactone) secretion, which suppresses the growth of neighbouring plants [133]. In addition to that, the plant can repel the larvae of the insect when applied to cultured media containing these larvae. It has also been applied as a repellent to moths and fleas. Several studies have shown that *A. absinthium* and its essential oil possess acaricidal [134], insecticidal [135], and repellent properties against flies, fleas, mosquitoes, and ticks [136,137]. They showed that ethanolic *A. absinthium* extract displayed 100% inhibition against cattle tick (*Rhipicephalus microplus*) eggs hatching in vitro, proposing that the plant could serve as a substitute to commercially available synthetic acaricides. Consistently, alcoholic *A. absinthium* extracts exerted high antifeedant activity rate on *Leptinotarsa decemnlineata*, *Rhopalosiphum padi* [138]. *A. absinthium* essential oils were reported to cause toxicity to adults of granary weevil *Sitophilus granarius* L. (Coleoptera). Additionally, *A. absinthium* essential oil exerted toxic effect on adult *R. dominica*, a stored product pest, with an LC_50_ value of 18.23 µL/L and LC_90_ value of 41.74 µL/L. The essential oils from *A. absinthium* showed significant fumigant activity on *S. littoralis* (a furthermost hazard pests of crops) with an LC_50_ value of 10.59 µL/L and LC_90_ value of 17.12 µL/L [27]. Govindarajan et al. [139] registered the larvicidal action of *A. absinthium* essential oils against *Culex quinquefasciatus*, *Culex tritaeniorhynchus*, *Anopheles subpictus*, *Aedes albopictus*, *Anopheles stephensi*, and *Aedes aegypti*. Few pharmacological effects of *A. absinthium* related to infectious diseases are shown in Table 5. Some of the mechanisms of action related to these activities are shown in Figure 2.

## 5. Pharmacokinetics of and Stability of *A. absinthium* Components

The artemisinin compounds are mainly converted into dihydroartemisinin (DHA) directly after its absorption and are thus transformed to inactive metabolites by hepatic cytochrome P-450 and other systems for enzymes and the elimination half-life of DHA was about 45 min. The level of conversion to DHA varies between artemisinin derivatives as artemisinin itself is not metabolized to DHA, while artesunate is rapidly hydrolyzed to DHA in few minutes. Whereas arteether and artemether are more gradually converted to DHA [142]. The thujone metabolism was studied in vitro in mouse, rat, and human liver cells and in vivo in rabbits, mice, and pigs. Two neutral urinary metabolites have been reported as 3-β-hydroxy-α-thujane and 3-β-hydroxy-β-thujane following oral administration of a mixture of α- and β-thujone at a dose level of about 650–800 mg/kg body weight to male rabbits [143]. α-Thujone was rapidly metabolised to 7-hydroxy-α-thujone, 4-hydroxy-α-thujone, 4-hydroxy-β-thujone, two other hydroxy-thujones and 7,8-dehydro-α-thujone by mouse hepatic microsomes [80]. Jiang et al. [144] as well as Abass et al. [145] stated that cytochrome P450 2D6 (CYP2D6) and CYP3A4 were the most effective cytochrome P450 (CP450) enzymes, generating 7-hydroxy-α-thujone, 4-hydroxy-thujone, suggesting that α-thujone is a liver blood–dependent compound. Artemisinin and artesunate can be administered in various dosing routes, including intramuscular (i.m.), rectal, intravenous (i.v.), and oral, while artemether can be used by i.m., oral, or rectal route. Medhi et al. [146] documented that the high first-pass metabolism is the key reason for poor oral bioavailability of artemisinin, artesunate and artemether, whereas artemether reaches peak levels in 2–6 h and artesunate reaches peak levels in only few minutes. They revealed that artesunate and artemether possess a moderate plasma protein binding ranging from 43 to 81.5 percent. Artesunate, as well as artemether, are thoroughly metabolized and converted to DHA with a plasma half-life of 1–2 h. Artenimol is another artemisinin metabolite [147]. Morris et al. [148] indicated that i.v. administration of artesunate yields a significantly higher C_max_ than that observed with any other route of administration. They reported that the average clearance values of artesunate and DHA are 2–3 L/kg/hr and 0.5–1.5 L/kg/hr, respectively, with the approximate volume of 0.1–0.3 L/kg for artesunate and 0.5–1.0 L/kg for DHA, whereas the i.m. administration of artesunate showed high bioavailability. Intramuscular artesunate shows similar pharmacokinetics to IV artesunate, however, it produces lower C_max_, higher volume of distribution, and longer half-life values for artesunate, and longer T_max_ values for DHA than IV administration [149]. Navaratnam et al. [150] recorded the pharmacokinetic parameters of rectal artesunate administration. They reported that rectal artesunate was similar to those obtained with oral administration, but artesunate T_max_ is delayed and its half-life is extended. Moreover, the population pharmacokinetic analyses of artesunate, as well as DHA after oral and rectal administration of artesunate, indicate that weight and pregnancy are important indicators of the pharmacokinetics of DHA following artesunate administration [148].

## 6. Combination Therapy of *A. absinthium* and Its Related Compounds with Other Drugs

Combination chemotherapies are now documented to relieve severe diseases, including malignancies, immune deficiency syndrome, lung tuberculosis, and several protozoa to facilitate improved therapeutic efficacy [151,152]. Notably, Batiha et al. [2] reported that the combined application of *A. absinthium* extract with diminazine aceturate and atovaquone showed additive and synergetic effects against *Babesia* and *Theileria* parasites in vitro and in vivo. Furthermore, World Health Organization (WHO) recommends artemisinin as well as artemether as a combination therapy with a generic antimalarial drug (lumefantrine) as the first-line antimalarial treatment in more than 50 countries suffering from chloroquine-resistant malaria [153]. The combination treatment of artemisinin with quinine and artemisinin with curcumin also demonstrated in vitro and in vivo synergistic effects toward malaria [154]. In addition to that, artesunate/pyronaridine, artesunate/amodiaquine, dihydroartemisinin/piperaquine, artesunate/mefloquine, and artesunate/sulfadoxine/pyrimethamine combination therapies have recently been approved for the treatment of artemisinin-resistant malaria [153].

## 7. Side Effects and Contraindications

**Figure 3:**
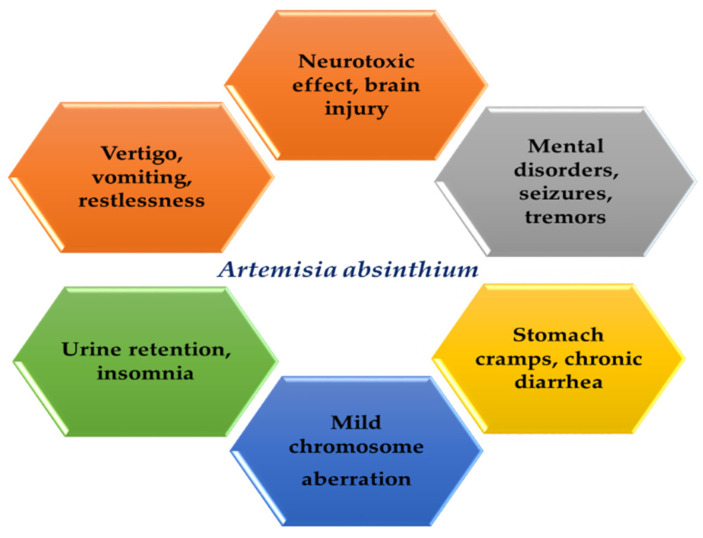
Schematic representation of different side effects of *A. absinthium* and its related compounds.

Lachenmeier et al. [61] reported that long-term administration of *A. absinthium* leads to some neurotoxic effect due to the presence of thujone and its analogues. Additionally, McGuffin et al. [155] observed that long-duration use of the essential oil obtained from *A. absinthium* may cause toxicity absinthism-mental disorder in humans with clinical manifestations which include convulsions, sleeplessness, and hallucinations. The adverse side effects may include stomach cramps, brain injury cramps, vertigo, vomiting, nausea, insomnia, restlessness, urine retention, seizures, and tremors [156]. Many toxicity studies were performed with thujone on experimental animals and revealed that it leads to dose-dependent toxic action [61]. The no-effect level (NOEL) has been reported to be in the range between 5 and 12.5 mg/kg body weight/day. Additionally, Juteau et al. [96] indicated that the essential oil obtained from *A. absinthium* contains high concentrations of thujyl alcohol, thujone, and other terpene-derivatives, which causes neurotoxicity at high concentrations. Thujone is an antagonist of Gamma-aminobutyric acid (GABA_A_) receptor that displays an epileptic-like convulsion by rapidly regulating the GABA-gated chloride channel [157]. Intraperitoneal injection of thujone causes convulsion in mice and the action was blocked by intraperitoneal injection of diazepam or phenobarbital [143]. Additionally, Rivera et al. [158] demonstrated a remarkable inhibition of the GABA_A_ receptor recruitment caused by acute stress after intracerebroventricular injection of α-thujone, and this may be because of α-thujone being acted to block the benzodiazepine binding site or other sites of GABA_A_. However, the formation of GABA_A_ receptors is a potential substrate to α-thujone than to other constitutive receptors. β-Thujone derivative (diastereoisomer α-thujone) also displays behavioral action [1]. Consistently, long-term treatment with high doses of *A. absinthium* leads to mild chromosome aberration [159]. *A. absinthium* essential oil is avoided in pregnant females, breastfeeding mothers, and individuals with hyperacidity, peptic ulcer patients, and individuals with allergy. Additionally, high dose administration of the plant can cause central nervous system disorders, intestinal cramps, vomiting, dizziness, and headaches. *A. absinthium* stimulates the remarkable blockage of acetylcholinesterase activity. Thus, this may be the main cause of chronic diarrhea in some situations because of increased acetylcholine concentration required to stimulate muscarinic receptors in the duodenum [160]. Figure 3 represented the side effects of *A. absinthium* and its related compounds.

## 8. Conclusions

This review examines the medicinal and side effects of *A. absinthium*. *A. absinthium* is a remarkable plant of the genus *Artemisia* that is commonly referred to as wormwood in the UK and absinthe in France. *A. absinthium* leaves were of great importance in botany and pharmaceuticals and are used in folk medicine against various diseases. It possesses antifungal, neuroprotective, insecticidal, antimicrobial, anthelmintic, acaricidal, antimalarial, antidepressant, and hepatoprotective activities. Previous reports documented that long-term use of *A. absinthium* leads to some neurotoxic effects due to the presence of thujone and its analogues. Administration of high dose of *A. absinthium* can cause central nervous system disorders, intestinal cramps, vomiting, dizziness, and headaches. *A. absinthium* essential oil is contraindicated in pregnant females, nursing mothers, and individuals with allergy, hyperacidity, and peptic ulcer patients.

## Figures and Tables

**Table 1:** Scientific classification of *Artemisia absinthium*.

Taxonomy	
Kingdom	Plantae
Division	Magnoliophyta
Class	Magnoliopsida
Order	Asterales
Family	Asteraceae
Genus	*Artemisia L*- sagebrush
Species	*absinthium*

**Table 2:** International Union of Pure and Applied Chemistry (IUPAC) name, and chemical structure of bioactive molecules isolated from *A. absinthium*.

Compound	Class of Compound	IUPAC Name	Chemical Structure
Artemisinin	Endoperoxide-containing sesquiterpene lactone	(3*R*,5a*S*,6*R*,8a*S*,9*R*,12*S*,12a*R*)-Octahydro-3,6,9-trimethyl-3,12-epoxy-12*H*-pyrano[4,3-j]-1,2-benzodioxepin-10(3*H*)-one	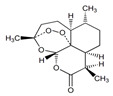
α-Thujone	Bicyclic monoterpene ketone	(1*S*,4*R*,5*R*)-4-Methyl-1-(propan-2-yl)bicyclo[3.1.0]hexan-3-one	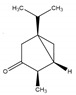
β-Thujone	Bicyclic monoterpene ketone	(1*S*,4*S*,5*R*)-4-Methyl-1-propan-2-ylbicyclo[3.1.0]hexan-3-one	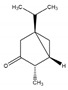
Bornyl acetate	Acetate ester of borneol, the bicyclic monoterpene	(4,7,7-Trimethyl-3-bicyclo[2.2.1]heptanyl) acetate	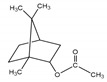
4-Terpineol	An isomer of the monoterpene alcohol, terpineol	2-(4-Methylcyclohex-3-en-1-yl)propan-2-ol	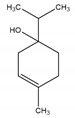
Camphene	Bicyclic monoterpene	2,2-Dimethyl-3-methylidenebicyclo[2.2.1]heptane	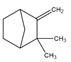
Chamazulene	A bicyclic unsaturated hydrocarbon. It is an azulene derived from sesquiterpenes	7-Ethyl-1,4-dimethylazulene	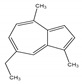
Cadinene	Bicyclic sesquiterpenes	(1*S*,4a*R*,8a*S*)-4,7-Dimethyl-1-propan-2-yl-1,2,4a,5,8,8a-hexahydronaphthalene	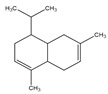
Myrcene	Alkene natural hydrocarbon, classified as a monoterpene	7-Methyl-3-methylene-octa-1,6-diene	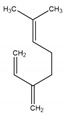
*trans*-Sabinyl acetate	Oxygenated monoterpene	[(1*R*,3*S*)-4-methylidene-1-propan-2-yl-3-bicyclo[3.1.0]hexanyl] acetate	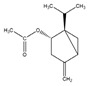
Guaiazulene	Bicyclic sesquiterpene, azulene derivative	1,4-Dimethyl-7-isopropylazulene	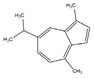
γ-Terpinene	Monoterpene	1-methyl-4-propan-2-ylcyclohexa-1,4-diene	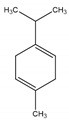
Linalool	Naturally occurring acyclic monoterpene alcohol	(3*R*)-3,7-dimethylocta-1,6-dien-3-ol	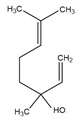
Camphor-	Terpenoid with the chemical formula C_10_H_16_O	1,7,7-Trimethylbicyclo[2.2.1]heptan-2-one	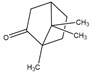

**Table 3:** Traditional uses of *A. absinthium*.

Geographical Location	Traditional Use	Part Used	Ref.
Brazil	Used for the treatment of digestive discomforts	*Artemisia absinthium* tea	[30]
Italy	Used an anthelmintic, digestive, antiemetic, antiparasitic, antihypertensive, and to relieve tendonitis	Leaves and aerial parts	[18]
Tunisia	Antimalarial	Aerial parts	[31]
Iran	Antimicrobial, diuretic, anthelmintic, choleretic, digestive.	Aerial parts	[32]
Pakistan	Used for fever treatment and as an anthelmintic for children.	Whole herb	[33]
Croatia	Digestive	Aerial parts	[34]
France	Antibacterial, appetite stimulant, antipyretic, emmenagogue, anthelmintic.	Aerial parts	[1]
China	Used to treat cancers, hepatic disorders, neurodegenerative diseases, acute bacillary dysentery.	Aerial parts	[35]
Cuba	Antimalarial	Whole herb	[1]
Western Europe	Stomach medicine useful for gastric pain, a cardiac stimulant, a restorative of declining mental functions.	Aerial parts	[36]
Bosnia and Herzegovina	Infusion used for gastrointestinal ailments, stomachache; decoction used for stomachache.		
Turkey	Used to treat stomach ache, as an appetizer, an abortive, blood depurative, diabetes, tuberculosis, antihypertensive, antimalarial, applied to wounds, antipyretic.	Aerial parts and leaves	[37]

**Table 4:** The pharmacological activity of *A. absinthium* and its related compounds.

Activities	Bioactive Compound	Mechanism of Action	Ref.
Antioxidant	Phenolic compounds and flavonoids	Reduction of lipid peroxidation level, decreasing TBARS level and the recovery of endogenous antioxidant (SOD, GSH)	[7]
Immuno-modulatory activity	Polysaccharides	Initiation of Th1 response and activation of NO synthesis	[56]
Wound Healing activity	β-thujone and β-pinene	Free radical scavenging activity	[24]
Neuroprotective	Combination of phytochemical compounds	Anticholinesterase activity	[58]
Antidepressant effects	Combination of phytochemical compounds	Inhibition of MAO, suppression of depression, inhibition of selective serotonin reuptake	[53]
Hepatoprotective Effects	Thujone	Suppression of liver microsomal drug-metabolizing enzymes, free radical scavenging activity, calcium channels blockage	[35,60]
Hypoglycaemic Effect	Thujyl alcohol, α- and β-thujones, azulenes, cadinene, bisabolene, sabinene, phellandrene, pinene	Stimulating AMPK, which mainly activated the translocation of insulin-stimulated GLUT4 to the cell surface	[69]
Anti-inflammatory	5,6,3′,5′-tetramethoxy 7,4′-hydroxyflavone, cardomonin, caruifolin D	Suppressing the proinflammatory mediators expression (iNOS, PGE(2), NO, COX-2, NF-kB) in LPS-stimulated RAW 264.7 cells and BV2 cells	[74,75,76]
Antitumor activity	Chlorogenic acid, artesunate, dihydro artesunate, artemisetin	Activation of the MEK/ERK pathway, activates the mitochondrial pathway of caspase activation, stimulate cell apoptosis	[41,42,43,44]
Antipyretic and analgesic activities	22-dien-3 Bat, 24-β-ethyl *p*-cholesta-7	Nicotinic and muscarinic action	[79]
Renal Effect	α- and β-thujones	High contents of total phenolic compounds and flavonoids as well as antioxidant effect of wormwood extract	[67]
Antiulcer and digestive activities	Bitter substances, essential oils	Enhancing the bile production and secretion	[23]

**Table 5:** The pharmacological activity of *A. absinthium* related to infectious diseases.

Activities	Bioactive Compound	Mechanism of Action	Ref.
Antibacterial Activity	Essential oil	Suppressing the biosynthesis of proteins, RNA, DNA and polysaccharide in the bacterial cells	[98]
Anthelmintic Activity	α-and β-thujones	Decrease juvenile (L3) larval motility and development of egg of *Ascaris suum* in an in vitro model	[126]
Anti-fungal Activity	Essential oil	High contents of total phenolic compounds and flavonoids	[94,121]
Antiprotozoal Activity	Essential oil, flavonoids, artemisinin	Stimulation of both heme and mitochondrial-mediated degradation cascade, inhibit *Pf*ATP6, inhibiting LDH, SAMS, PyrK, SpdSyn, OAT enzyme activities	[116,120,140]
Insecticidal Effect	Essential oil	Toxicity to adults of granary weevil *Sitophilus granarius* L., resulting in 80–90% mortality rate of these insects	[133,134,135,136]
Antiviral Activity	Several bioactive compounds	Inhibiting the integrase enzyme from human immunodeficiency virus (HIV-1) from connecting the DNA from the host cell with the reversibly transcribed viral DNA	[141]

## Abbreviations

*A. absinthium*: *Artemisia absinthium*; IUPAC: International Union of Pure and Applied Chemistry; HIV-1: human immunodeficiency virus; HBV: hepatitis B virus; HDP: heme detoxification protein; *S. aureus*: *Staphylococcus aureus*; *B. subtilis*: *Bacillus subtilis*; *B. cereus*: *Bacillus cereus*; *Sal. typmiruim*: *Salmonella typmiruim*; *E. coli*: *Escherichia coli*; *P. aeruginosa*: *Pseudomonas aeruginosa*; *T. brucei*: *Trypanosoma brucei*; *L. infantum*: *Leishmania infantum*; *A. fumigatus*: *Aspergillus fumigatus*; *Pf*TCTP: *Plasmodium falciparum* controlled tumor protein; MIC: minimum inhibitory concentration; LDH: L-lactate dehydrogenase; SAMS: S-adenosyl-methionine synthetase; cGMP: cyclic guanosine-3′:5′-monophosphate; PyrK: pyruvate kinase; SpdSyn: spermidine synthase; OAT: ornithine aminotransferase; DPPH: 2,2-diphenyl-1-picrylhydrazyl; IBD: Inflammatory bowel disorders; UC: ulcerative colitis; CD: Crohn’s disease; COX-2: cyclooxygenase-2; NO: nitric oxide; NOS: nitric oxide synthase; TNF-α: tumor necrosis factor-α; NF-*κ*B: nuclear factor- kappa B; LPS: lipopolysaccharide; NOEL: no-effect level; GABA: Gamma-aminobutyric acid; AChE: acetylcholinesterase enzymes; AMPK: adenosine monophosphate-activated protein kinase; GLUT4: insulin-stimulated glucose transporter type 4; MAO: monoamine oxidase; SOD: superoxide dismutase; GSH: glutathione; iNOS: inducible NO synthase; PGE(2): prostaglandin E(2); MEK/ERK: mitogen-activated protein kinase kinase/extracellular signal-regulated kinase; Th1: T helper cell 1; GABA_A_: Gamma-aminobutyric acid; TBARS: thiobarbituric acid reactive substances; H_2_O_2_: hydrogen peroxide; DCs: dendritic cells; IgA: immunoglobulin A; LH: luteinizing hormone; MIC: minimal inhibitory concentration; MBC: minimal bactericidal concentration; DNA: deoxyribonucleic acid; *A. absinthium*-Ag NPs: *A. absinthium*-silver nanoparticles; DHA: dihydroartemisinin; CYP2D6: cytochrome P450 2D6; CP: cytochrome P450; WHO: World Health Organization; *Kl. Oxytoca*: *Klebsiella oxytoca*; MRSA: methicillin-resistant *S. aureus*.
